# Polyphilic Interactions as Structural Driving Force Investigated by Molecular Dynamics Simulation (Project 7)

**DOI:** 10.3390/polym9090445

**Published:** 2017-09-14

**Authors:** Christopher Peschel, Martin Brehm, Daniel Sebastiani

**Affiliations:** Institute of Chemistry, Martin-Luther-Universität Halle-Wittenberg, von-Danckelmann-Platz 4, 06120 Halle, Germany; christopher.peschel@chemie.uni-halle.de (C.P.); martin.brehm@chemie.uni-halle.de (M.B.)

**Keywords:** force field, polyphilic, DPPC, membrane, fluorophilic, bolapolyphile, perfluorinated, CHARMM, lipid bilayer

## Abstract

We investigated the effect of fluorinated molecules on dipalmitoylphosphatidylcholine (DPPC) bilayers by force-field molecular dynamics simulations. In the first step, we developed all-atom force-field parameters for additive molecules in membranes to enable an accurate description of those systems. On the basis of this force field, we performed extensive simulations of various bilayer systems containing different additives. The additive molecules were chosen to be of different size and shape, and they included small molecules such as perfluorinated alcohols, but also more complex molecules. From these simulations, we investigated the structural and dynamic effects of the additives on the membrane properties, as well as the behavior of the additive molecules themselves. Our results are in good agreement with other theoretical and experimental studies, and they contribute to a microscopic understanding of interactions, which might be used to specifically tune membrane properties by additives in the future.

## 1. Introduction

Self-assembling compounds are of huge interest for various fields of research [1,2]. On the one hand, nanosized materials can be constructed by tuning the properties of their precursors, and on the other hand, biochemical materials such as lipid bilayers are broadly investigated [3]. In the context of amphiphilic molecules, polyphilic molecules possess even greater complexity within one molecule. As the name suggests, polyphilic molecules combine many philicities in one molecule and therefore have various special properties [4,5,6,7,8,9]. Within the framework of lipid bilayers, the well-known amphiphilic molecules as well as polyphilic molecules can be used as drug delivery agents [10,11]. The insertion of trans-membrane molecules can cause effects such as stretching or compression of the bilayer, which was recently shown for polyphilic molecules [12,13,14,15,16,17]. Fluorocarbon compounds are also of interest as additives; they have recently been used for in vitro synthesis of membrane proteins [18] and for influencing the metabolism of rats [19,20].

In contrast to experiments, which usually determine macroscopic properties of the system, molecular dynamics simulation can provide a molecular insight into the lipid bilayer and into the behavior of additive molecules within. A lipid bilayer can be considered as consisting of two parts. The head-group regions of the lipid molecules, which are significantly polar, comprise the hydrophilic part of the membrane. The remainder of the lipid molecules consists of hydrocarbon chains, which are non-polar and therefore lipophilic. There exist numerous lipid bilayer-forming agents. Dipalmitoylphosphatidylcholine (DPPC) is a typical example; on the one hand, it is extensively studied experimentally [14,21,22,23,24,25], while, on the other hand, it is well-covered in force-field support [26,27,28].

Inserting additives of high polarity into such a bilayer would most likely result in incorporation into the polar head-group region. Agents that are rather non-polar, on the other hand, are expected to concentrate in the middle of the lipid bilayer, at the largest distance to the head-group region. The question regards what happens when fluorinated agents are added into the membrane. Fluorinated molecules are known to show neither hydrophilic nor lipophilic behavior. Without deep investigation of these systems, one cannot exactly predict the behavior. This question has recently been investigated within numerous experiments and simulations, and it is extensively studied within this project [13,14,29,30,31,32].

In this review article, we summarize our efforts to address the influence of polyphilic molecules on lipid bilayer properties and the characterization of their behavior itself by the usage of force-field molecular dynamic simulations in the framework of the research consortium “Forschergruppe 1145”.

## 2. Computational Details

**Figure 1:**
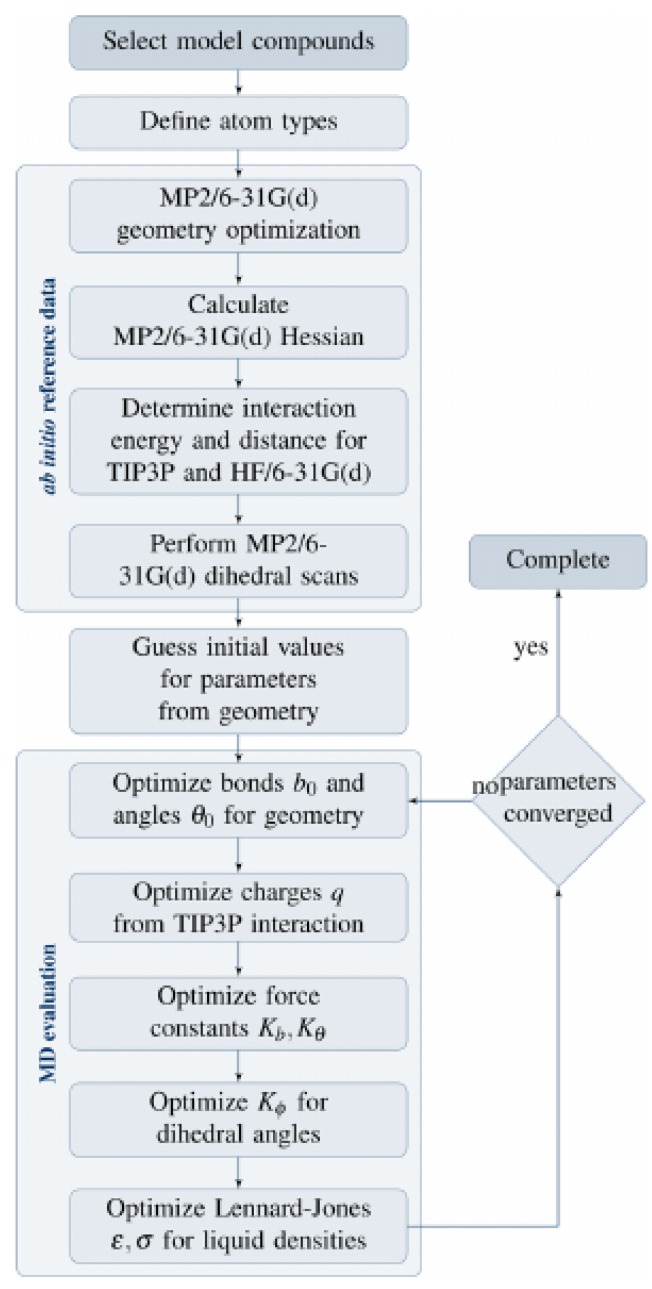
Parametrization scheme for the CHARMM force field as applied in this work.

Within this project, adequate force-field parameters for the interaction between lipid molecules and perfluorinated alkanes have been developed. These parameters have been developed for usage in the framework of the CHARMM force field [33]. The scheme of parameter generation is shown in Figure 1. For a more detailed description, we refer to the original research article [34].

To investigate the influence of additive molecules on DPPC bilayers, many different molecular dynamics simulations have been performed. An overview of the systems investigated within this project is given in Table 1.

The plane of the lipid bilayers was chosen to have its normal vector in the Z direction. Every simulation was performed in a periodic box and the lipid bilayer slabs were separated by water layers of about a 4 nm diameter. Additive molecules were inserted directly in the middle of the DPPC bilayer. The system’s sizes and equilibration times varied for the different simulations, and we refer for further details to the sections of this review article and to the original publications [30,31,32].

The simulations were performed with the program package NAMD 2.9 [35], within the NpT ensemble, using a Langevin thermostat with a damping parameter of 1.0 ps to control the temperature. All calculations were kept at atmospheric pressure by the Langevin piston Nosé–Hoover method (oscillating period of 200 fs; damping time of 100 fs). The CHARMM force field [33] complemented by our own developed parameters [34] was used. The time step of the different simulations varied from 1.0 to 2.0 fs. The bond lengths were constrained using the SHAKE algorithm. The cutoff distance for the non-bonded interactions was set to 1.5 nm, with a switching distance of 1.2 nm. It is known that electrostatic long-range interactions are of particular importance for this kind of system [36]; thus, we applied the particle mesh Ewald (PME) method to handle long-range interactions. The initial atomic velocities were randomly generated from a Maxwell–Boltzmann distribution centered at the respective simulation temperature. The TIP3P water model was used to solvate the system [37,38]. Data analysis of the trajectories were performed by using VMD plugins [39], the python module MDAnalysis [40], the freeware program package TRAVIS [41], and our own code. The plots in this article have been created with xmgrace [42], TRAVIS [41] and Gnuplot [43].

## 3. Results

### 3.1. Perfluoroalkane Force Field for Lipid Membrane Environments

**Figure 2:**
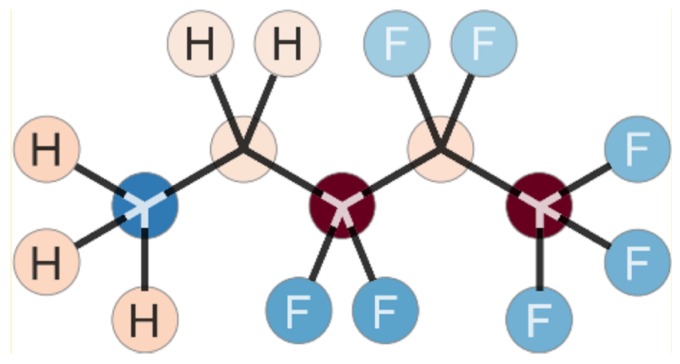
Illustration of the different atom types for parametrization. Four new atom types have been introduced: carbon atoms in a CF${}_{3}$ group (dark red circle on the right side), carbon atoms in a CF${}_{2}$ group (beige-colored circle on the right side), and respectively for each group, separate types of fluorine atoms (light blue and blue circles on the right side).

When performing force-field molecular dynamics simulations, the choice of a force field for the bonded and non-bonded molecular interactions is crucial. As there existed no adequate force field-combining lipid molecules and fluorinated molecules, the parameters covering these interactions have been developed within this project [34]. The CHARMM force field is known to be fast and precise for a broad range of molecules, especially lipid membranes and their single lipid molecules [26,44]. Therefore, it seemed reasonable to extend this force field by the missing parameters for fluorinated molecules (see Figure 2). This was done by using the CGenFF method [33]. This method shares the same basic idea as for the CHARMM force-field parameter generation, but it is described in more detail in literature.

First of all, the geometrical structure of the model compound was optimized by a quantum chemical calculation at the MP2/6-31G(d) level. Within this optimized geometry, the equilibrium values for the bond lengths, angles, and dihedral angles together with their phases and their multiplicities are accessible. Afterwards, the partial charges on all the atom types were obtained from an iterative scheme and comparison to quantum chemical calculations at the HF/6-31G(d) level of theory. The third step consisted of the calculation of the force constants from vibrational frequencies obtained from normal-mode analyses with an empirical scaling factor of 0.94327 (for the for MP2/6-31G(d) level of theory) [33,45,46] to account for anharmonicity. In the next step, the energy profiles of the torsional angles were analyzed using a relaxed surface scan by distorting the minimum-energy geometry. Afterwards, the free parameters in the dihedral energy term were fitted to reproduce the MP2/6-31G(d) energy profile as well as possible.

**Figure 3:**
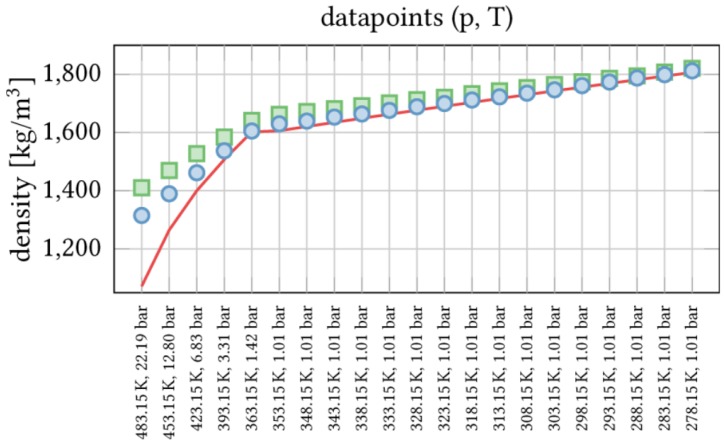
Computed densities for perfluorinated octane and different combinations of pressure and temperature. The optimized parameters found in this work (●) reproduce the experimental values (/) better than the original CGenFF parameters (◼).

Special care had to be taken for the calculation of the van der Waals parameters [34], as they are crucial for the interactions between lipid bilayers and fluorophilic molecules. The parameters were fitted to experimental densities of liquid perfluoroalkane with an average relative error of 0.6%. A broad range of both pressure and temperature were taken into account, producing very good results (see Figure 3). The interaction between the different atom types was described by the Lorentz–Berthelot mixing rules [47,48].

The force-field parameters have been benchmarked for interaction energies with water, pure liquid densities, miscibility with alkanes, and thermodynamic properties (heat of vaporization, heat capacity, thermal expansion coefficient, static dielectric constant, and viscosity). The results are in excellent agreement with experimental data and therefore seem to be very accurate in accounting for the subtleties in lipid membrane fluorophilic interactions.

### 3.2. Influence of Small Fluorophilic and Lipophilic Organic Molecules on DPPC Bilayers

Perfluorinated *n*-alkanes represent an interesting and special class of molecules because of their specific and unusual properties. As a result of the special nature of the C–F bond, they are considerably more hydrophobic than lipids, but they are not lipophilic either, which allows for interesting applications both in materials science and biochemistry. Perfluorinated compounds (PFCs) have numerous applications for medical purposes as oxygen-carrier fluids [49,50], in the purification or polymerization [49], and as lubricants [51].

Studies have found that PFCs cause alterations in cell membrane properties [52]. Molecules containing perfluorinated alkyl chains influence channel formation when they are added to a membrane environment [9], and they also affect the overall stability [53] and surface properties [22] of a lipid membrane. The incorporation of fluorinated surfactants in lipid bilayers greatly influences the chain order and permeability of liposomes [54]. Another interesting effect of the incorporation of fluorinated chains is their impact on liposome gel regarding liquid-crystalline phase-transition temperatures T${}_{m}$. Although the incorporation of fluorinated chains in liposome bilayers can increase the characteristic gel to the liquid-crystalline phase-transition temperature (T${}_{m}$), this effect is highly dependent on other structural features, which include the length, relative proportions and symmetry of the fluorinated segments in the bilayer [55,56].

**Figure 4:**
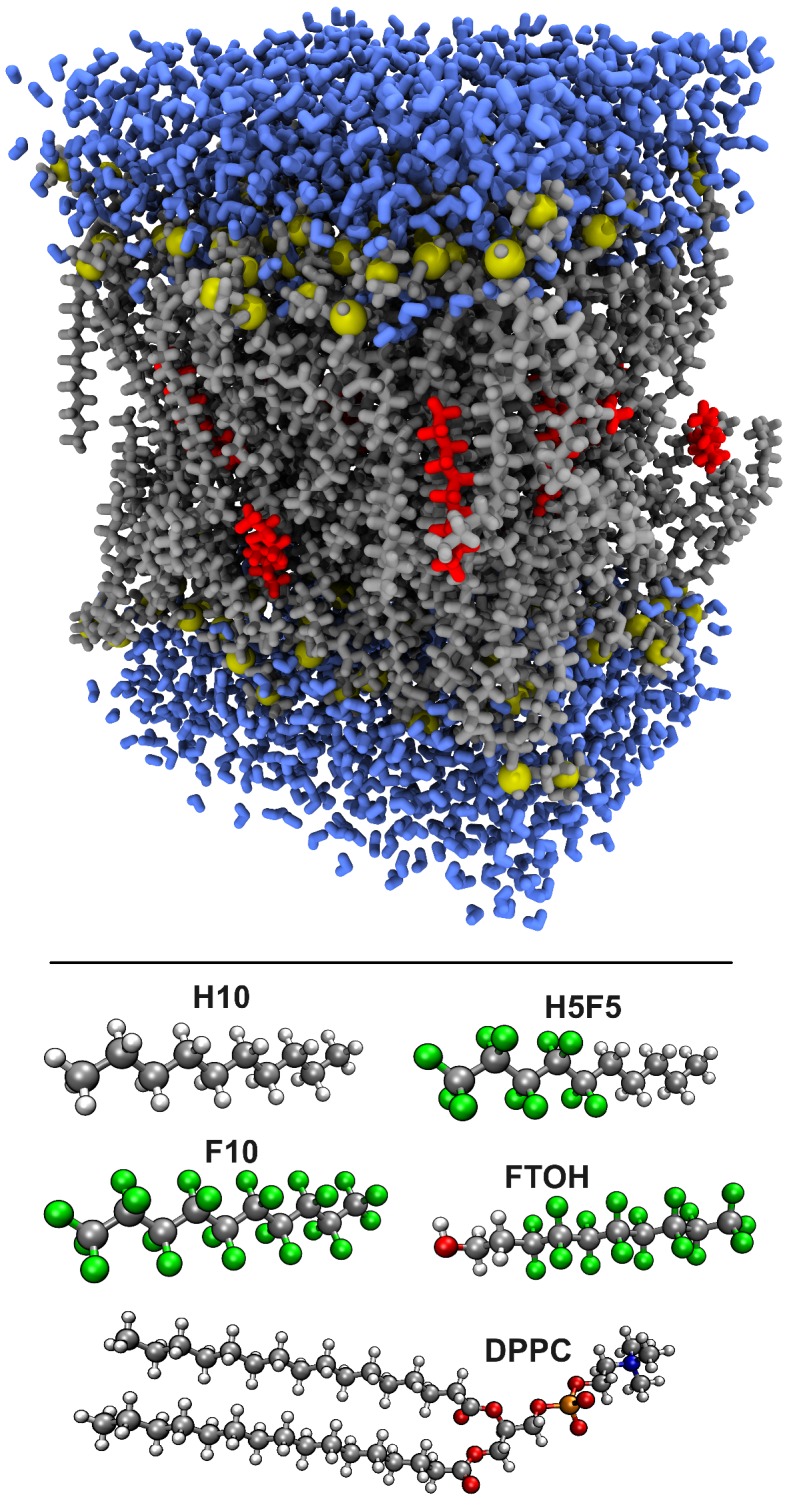
Upper panel: Snapshot of one of the simulation cells (gray: dipalmitoylphosphatidylcholine (DPPC) C/H/O atoms; yellow: DPPC N/P atoms; red: *n*-decane; blue: water). Lower panel: Molecular structures of the molecules involved in this study.

In order to investigate these effects, we used our recently developed perfluoroalkane force field [34] to perform molecular dynamics simulations of a series of additive molecules with different types of philicities inside a DPPC bilayer [31]. On the basis of these simulations, we investigate the effect of the additives on the structure and dynamics of the membrane. To elucidate the effect of the additives, we compared the simulations to a system with a pure DPPC bilayer without additive molecules. A snapshot of one of the simulation cells as well as the structures of the investigated additive molecules can be found in Figure 4.

**Figure 5:**
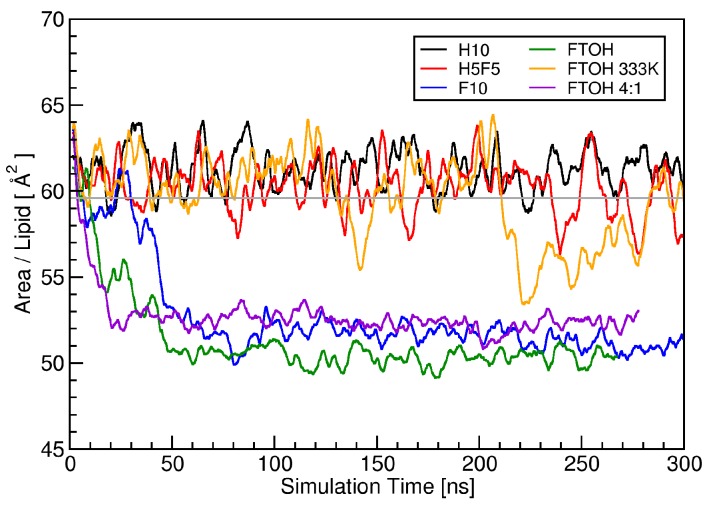
Temporal development of the lateral area per lipid during simulation runs. The gray line depicts the average value of the pure membrane.

**Figure 6:**
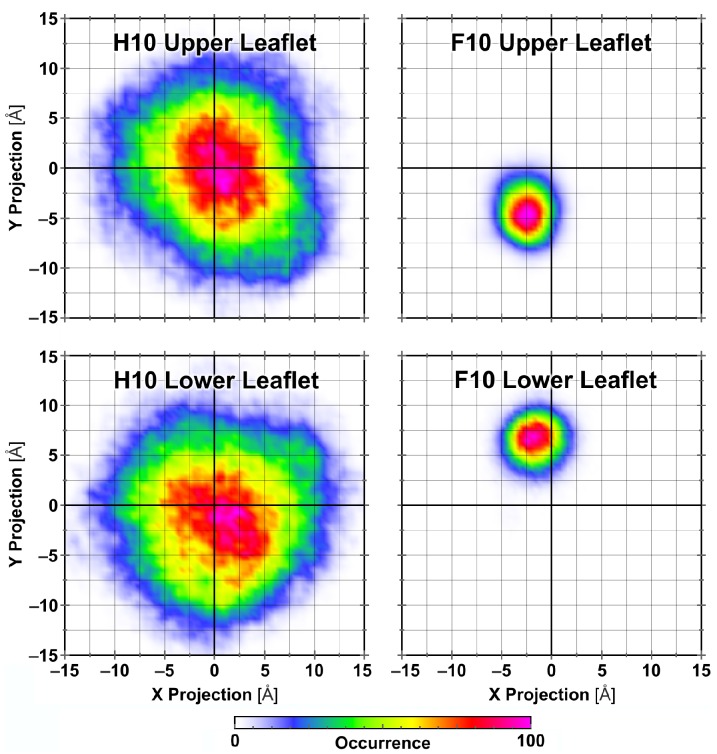
Projection of the dipalmitoylphosphatidylcholine (DPPC) chain tilting vectors (from α- to ω-carbon atom of carboxylic acids) into the X–Y plane for the H10 (**left**) and F10 (**right**) systems during the last 5 ns of the trajectories. Collective tail tilting (as on the right side) indicates gel phase [31].

It has been extensively discussed in literature that the lateral area per lipid can be a good measure of the order in DPPC bilayers, and that a significant change in the area per lipid can be an indication of phase transitions [57,58,59,60,61]. Therefore, we started our analysis by investigating the temporal development of the area per lipid in our simulation trajectories. On the basis of the temporal development of the area per lipid values (see Figure 5), we found that the addition of perfluoro-*n*-decane and fluorotelomer alcohol at 323K leads to a phase transition of the membrane (i.e., from liquid-crystalline to gel phase), whereas the addition of *n*-decane and partially fluorinated *n*-decane leaves the liquid-crystalline phase intact. In the gel phase, the lipid molecules are more ordered than in the liquid-crystalline phase. The gel phase is characterized by the collective tail tilting of the alkyl chains of the lipid molecules. By defining the vector from the α-carbon to the ω-carbon as the tail vector and projecting into the X–Y plane, it can be used to characterize the phase of the lipid bilayer. The liquid-crystalline phase showed a random distribution of the tail vectors, whereas the gel phase showed a pronounced collective tilting, aside from at the origin. This is shown in Figure 6. The addition of fluorotelomer alcohol at a slightly increased temperature of 333 K prevented the phase transition from occurring. These results are substantiated by the diffusion constants, which we calculated from the mean square displacements: The systems in the gel phase showed a significantly reduced diffusivity for both the DPPC and additive molecules (see Table 2).

The addition of non-fluorinated and partially fluorinated *n*-decane, on the other hand, led even to an increased DPPC diffusivity with respect to the pure bilayer. In these two systems, the additive molecules possess a very high diffusion constant, leading to the conclusion that they show almost no interactions to DPPC, and move freely in the center of the bilayer (like a lubricant between the layers).

By investigating the density profiles, we found that the phase transition of the membrane not only reduces the area per lipid, but increases the diameter of the membrane at the same time, such that the molecular volume of DPPC remains almost constant. While non-fluorinated and partially fluorinated *n*-decane is found mainly in the middle of the bilayer, perfluoro-*n*-decane penetrates significantly deeper into the membrane leaflet, which triggers a phase transition. Fluorotelomer alcohol is found almost exclusively inside the leaflet. The hydroxyl groups of fluorotelomer alcohol point to the outside of the bilayer in almost all cases. This is due to a very strong hydrogen bond between the hydroxyl group and the double-bound ester oxygen atoms in the head group of DPPC. The hydroxyl groups of the alcohol form an extended hydrogen-bond network with the DPPC ester groups, which chains these molecules together and significantly hinders the lateral diffusion of both DPPC and alcohol molecules. Interestingly, a slight increase in the temperature by only 10 K is sufficient to dynamically overcome this hydrogen-bond network (as shown by the increase of diffusion constants), despite that the hydrogen bonds are still strongly populated.

Concerning the conformations of the additive molecules, fluorotelomer alcohol is almost only present in the all-trans conformation with maximal spatial chain length (with the hydroxyl groups pointing towards the polar head groups of the membrane and the alkyl chains pointing inside the membrane). Perfluoro-*n*-decane also prefers the all-trans configuration. This effect reduces with partially fluorinated *n*-decane, which also assumes other configurations over some time. Finally, non-fluorinated *n*-decane has no preference for the all-trans configuration and assumes all possible conformations, best described as random-coil configuration. This is nicely in line with the finding of very weak interactions between *n*-decane and DPPC [33], as liquid bulk *n*-decane (without the influence of a membrane) also prefers a random-coil configuration.

### 3.3. Conformational Space of a Polyphilic Molecule with a Fluorophilic Side Chain Integrated in a DPPC Bilayer

Polyphilic molecules per definition combine many philicities in one molecule. This makes them especially interesting because the philicities oppose each other. As it is very hard to predict the exact behavior of these types of molecules, they have been investigated by many researchers lately [12,16,17,62,63,64,65]. They are known to modify the properties of membranes, but an insight on the molecular scale is hard to obtain from experiments.

**Figure 7:**
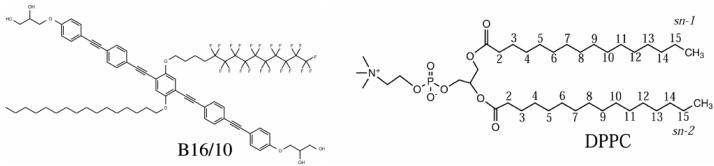
Chemical structure of the polyphilic molecule B16/10 and dipalmitoylphosphatidylcholine (DPPC) molecule considered in this work.

Therefore, we simulated one single bolapolyphile molecule (BP) in a DPPC membrane [30]. As an archetype of such polyphilic molecules, the bolapolyphile B16/10 was chosen. The B16/10 molecule possesses a phenylene ring backbone that is terminated with a glycerol group at both ends (see Figure 7). In the middle of the backbone, two side chains are attached—one of these is a perfluoro-*n*-alkane, while the other is a regular *n*-alkane. This molecular structure ensures a trans-membrane orientation of the backbone, yielding an anchor point for the alkane/perfluoroalkane chains at the center of the membrane. Therefore, the B16/10 molecule combines hydrophilic, lipophilic, fluorophilic, and aromatic parts in one molecule. Fluorophilic means that it has neither an affinity to polar molecules such as water, nor an affinity to lipophilic molecules such as alkanes.

The simulation setup consisted of a pre-equilibrated DPPC bilayer slab of 288 lipid molecules, enclosed by a water layer of roughly 10 Å, which summed up to a periodic simulation box of 95×95×68 Å. For our calculations, we chose the CHARMM force field [33,44], which comes with both an extensively tested support [26] for a broad range of lipids and a clear parametrization procedure. For the glycerol end groups, the parameters are available in literature [66,67]. Recently, we have presented parameters for perfluoroalkanes [34].

We performed molecular dynamics simulations in NpT ensemble with a time step of 1 fs in all cases; the trajectories were equilibrated for 5 ns and run for a total of 50 ns each (or 200 ns total). Additionally, one simulation with rigid bonds and a time step of 2 fs for 400 ns was performed to assess the diffusion. The mean square displacements showed a diffusion in good agreement with experimental values for the DPPC molecules [68].

We performed the simulations by starting from two different initial conformations in two different orientations. We chose the two low-energy conformations of the side chains that offerred a strong change in the overall conformation of the molecule: all-trans (x-shaped) and all-trans with the first dihedral at the central phenyl ring turned by 180${}^{\circ }$ (cross). Additionally, we started from two different orientations of the phenyl backbone, either directly perpendicular to the membrane plane, or tilted by an angle of about 15${}^{\circ }$. This is a typical angle found in experiments. As the polyphilic molecule is slightly longer than the membrane thickness, this angle also allows for the hydrophilic head groups to arrange next to the DPPC head groups. All the calculations were performed at atmospheric pressure, while the temperature was kept at 330 K. This temperature was just above the transition temperature of the membrane lipids from the gel state to the liquid-crystalline state [24,69], which is reproduced by the force field [26].

The conformational space of the side chains is the key to the understanding of their structure–function relationships. Experimentally, polyphilic molecules are known to integrate into a lipid bilayer membrane, despite that perfluoro-*n*-alkanes are not miscible with *n*-alkanes [25,70,71,72].

We observed a significant difference between the lipophilic and the fluorophilic side chains regarding their intra-membrane distribution. While the lipophilic groups remained membrane-centered, the fluorophilic parts tended to orient toward the phosphate head groups. This trend is important for understanding the influence of polyphilic agents on the properties of phospholipid membranes. From a fundamental point of view, our computed distribution functions of the side chains are related to the interplay of sterical, enthalpic, and entropic driving forces. Our findings illustrate the potential of rationally designed membrane additives, which can be exploited to tune the properties of phospholipid membranes.

**Figure 8:**
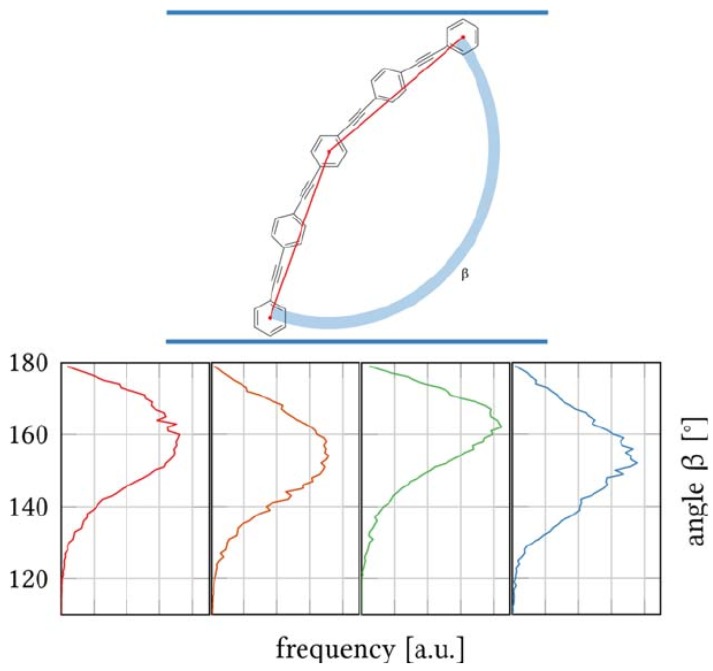
Top: Definition of the backbone bending angle β. Bottom: Distribution functions (histogram renormalized with 1/sinβ) for each of the four trajectories.

Figure 8 shows the distribution functions of the backbone bending angle β of the polyphilic molecule for each of the four independent trajectories. The distribution maxima vary between 150${}^{\circ }$ and 165${}^{\circ }$, and the tails extend up to 130${}^{\circ }$ and 145${}^{\circ }$. The variance observed for the different trajectories shows that full phase-space convergence had not yet been reached. Nevertheless, the similarity between the distribution functions indicates that the characteristics of the distribution functions are most likely realistic. A feature common to all the trajectories is the absence of conformations with angles β close to 180${}^{\circ }$ (despite the normalization with 1/sinβ). This indicates that the polyphiles are always bent when inserted into the lipid membrane. This bending is indicative of a slight mismatch between the length of the lipophilic core of the polyphile and the thickness of the DPPC membrane. This result might have implications for experiments in which the backbone is assumed to be straight in the analysis and interpretation of measurement results [73].

Our simulations show that the polyphile is indeed commensurate with the membrane, albeit a certain intramolecular bending and an inclination with respect to the membrane plane being observed, which is attributed to a slight size mismatch between the membrane and the lipophilic backbone of the molecule. We find an interesting difference in the orientational preferences between the perfluorinated side chain and the non-fluorinated alkane chain. The perfluorinated side chain orients along the lipophilic chains and towards the phosphate head groups of the DPPC molecules, while the alkane side chain remains mostly in the center of the bilayer. The self-diffusion for the lipids is in good agreement with experimental data.

Our simulations are a first step towards the understanding of the intra-membrane structure of polyphilic molecules with specifically designed side chains of different philicity. These molecules have the potential to enable a controlled modification of membrane properties such as water and ion permeability.

### 3.4. Characterization of Dynamical Behavior and Orientation of a Cluster of B16/10 Polyphiles in DPPC Membrane Environment

**Figure 9:**
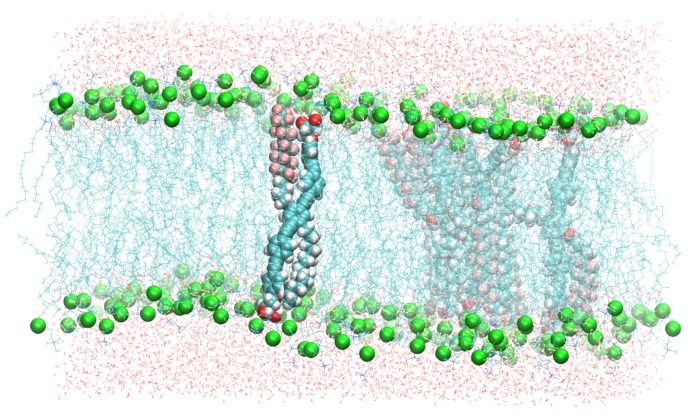
A snapshot of the simulated system containing six B16/10 molecules, 288 dipalmitoylphosphatidylcholine (DPPC) molecules, and 8756 water molecules. Periodic boundary conditions were used in all directions. Atoms of B16/10 molecules and phosphorus atoms of DPPC head groups are represented by solid spheres. Phosphorus atoms are green, fluorine atoms of B16/10 side chains are pink, and carbon atoms are cyan. All the lipid tails of DPPC are represented by cyan lines.

Subsequent to the last project, we extended the number of additive molecules within the bilayer to six in order to investigate whether the polyphiles cluster or distribute as single molecules in the membrane. The available experimental results indicate that the incorporation of BPs into gel-phase lipid (DPPC) bilayers leads to the formation of large BP domains within the membrane, and a separation into different lamellar species can be observed [12]. The thermal behavior of the lipid membranes was drastically altered upon BP incorporation, and several endothermic transitions above *T*${}_{m}$ of pure DPPC membrane occurred. In the liquid-crystalline phase, the BPs were homogeneously distributed in the membrane plane [12,16]. Our periodic simulation box of 98×98×68 Å contained 288 lipid molecules (144 per leaflet) and 6 B16/10 polyphiles (see Figure 7 of the previous section), and it was solvated by 8756 water molecules (modeled by the TIP3 force field). A snapshot of the system is presented in Figure 9.

The system was kept at a constant pressure of 1 bar and a constant specified temperature (isobaric–isothermal NPT ensemble) using a modified Nosé–Hoover method in which Langevin dynamics is used to control fluctuations in the barostat. The experimentally measured phase-transition temperature of DPPC membranes (gel phase to liquid-crystalline phase) was found to be between 313 and 315 K. It is experimentally observed that the presence of polyphile molecules increases this phase-transition temperature. In order to avoid a simulation in the gel phase, we set the simulation temperature to 335 K in this work. The system was simulated for 1 ms with a time step of 2 fs. The bond lengths were constrained using the SHAKE algorithm. For the purpose of comparison, a simulation of a pure DPPC membrane system was carried out under the same conditions.

A statistical analysis was carried out to characterize the dynamical and structural properties of B16/10 trans-membrane molecules inside a DPPC bilayer. In particular, we calculated the lateral diffusion coefficients of DPPC and B16/10 molecules [32] and investigated the axial location and orientation of B16/10 molecules as well as the internal structure of the B16/10 cluster. The results are compared to the pure DPPC membrane system, which itself is compared to experimental data [74].

The diffusion coefficients have been found to be in very good agreement to the experimental values. The side-chain orientation and backbone angle distribution resemble those of our former simulations [30]. In addition, we found that the perfluorinated side chains flip from one head-group region to the opposing region on a nanosecond time scale rather than remaining in the middle of the membrane.

**Figure 10:**
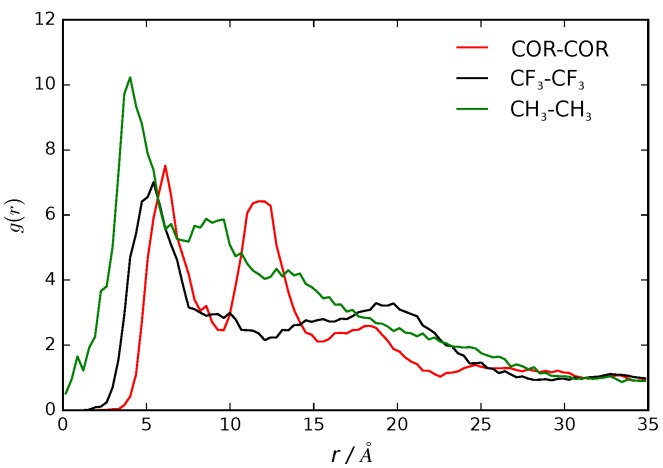
Radial distribution functions of the center of the central phenylene ring (COR), and CF${}_{3}$ and CH${}_{3}$ terminal groups of B16/10 molecules within the dipalmitoylphosphatidylcholine (DPPC) bilayer.

We calculated the pair correlation function, g(r), as the probability of finding a pair of B16/10 molecules at a distance r apart, relative to the probability expected for a completely uniform distribution at the same density [75]. The g(r) function of the center of mass for each central phenylene ring of a B16/10 molecule (COR-COR) and of its terminal groups (CH${}_{3}$ and CF${}_{3}$) is shown in Figure 10. We observed a clustering effect, indicated by the two-peak nature of the radial distribution function. The first peak at the 7 Å distance shows that two B16/10 molecules are directly adjacent to each other, as there is no space for interlaced molecules. Nevertheless, the lipid molecules force the B16/10 molecules to tilt a little away from a coplanar orientation and cause the observed distance of 7 Å. The second peak at 12 Å shows that a third B16/10 molecule forms a small cluster with the other two. As the second peak is not located at double the distance of the first, it can be deduced that the three molecules are not present in a linear arrangement, but rather, they form a triangle. Altogether, this shows that there is a correlation between the backbones of the B16/10 molecules, which form a small cluster, whereas there seems to be no correlation between the side chains aside from their pairwise orientation.

Our results give insights not only into the conformational preference of the B16/10 molecules inside the DPPC bilayer, but also on the dynamics of the B16/10 molecules in the DPPC bilayer environment. Within 1 ms of simulation, the stability of the membrane was maintained upon the insertion of the additive B16/10 molecules. The diffusion of the lipids of the membrane was nearly unchanged compared to a pure membrane, and B16/10 molecules moved only slightly slower than the lipids. For the intermolecular structure of the B16/10 molecules, a clustering effect could be observed in the RDFs. The difference in the side-chain orientation and configuration was also in good agreement with the simulation results we obtained previously [30]. In general, the conclusions drawn from the simulations are consistent with experimentally observed effects. We anticipate that these findings will be important for understanding the role of polyphile molecules in modulating and modifying lipid membrane properties.

## 4. Conclusions

In this review, we have discussed and summarized the results of a series of research projects that have emerged from sub-project TP 7 within the research consortium “Forschergruppe 1145”. First of all, we have built up the basis for accurate force-field molecular dynamics simulations of polyphiles within lipid membranes by the construction of an adequate all-atom force field. With these force-field parameters, we performed three sets of simulations on lipid membranes containing additive molecules at different temperatures. From these simulations, we investigated the influence of the additives on the membrane properties, as well as the behavior of the additive molecules themselves on a microscopic scale. Our results are in good agreement with the results from other sub-projects and corresponding collaborations (TP1-6, Tschierske, Binder, Kressler, Blume, Saalwächter, and Bacia). The results give a first insight into the behavior of polyphilic molecules inside lipid membranes and give important information for the tuning of molecules to particularly influence the membrane properties, which could be beneficial for broad fields of chemistry and biochemistry.

## Figures and Tables

**Table 1:** Systems studied [30,31,32].

System	Composition	Mixing Ratio	Simulation Temp. [K]	Simulated Time [ns]
Pure	72 DPPC 2189 H${}_{2}$O	—	323	69.3
H10	72 DPPC 2189 H${}_{2}$O 12 CH${}_{3}$(CH${}_{2}$)${}_{8}$CH${}_{3}$	6:1	323	311.2
H5F5	72 DPPC 2189 H${}_{2}$O 12 CH${}_{3}$(CH${}_{2}$)${}_{4}$(CF${}_{2}$)${}_{4}$CF${}_{3}$	6:1	323	301.0
F10	72 DPPC 2189 H${}_{2}$O 12 CF${}_{3}$(CF${}_{2}$)${}_{8}$CF${}_{3}$	6:1	323	301.5
FTOH	72 DPPC 2189 H${}_{2}$O 12 CF${}_{3}$(CF${}_{2}$)${}_{7}$(CH${}_{2}$)${}_{2}$OH	6:1	323	268.9
FTOH 333 K	72 DPPC 2189 H${}_{2}$O 12 CF${}_{3}$(CF${}_{2}$)${}_{7}$(CH${}_{2}$)${}_{2}$OH	6:1	333	301.9
FTOH 4:1	72 DPPC 2189 H${}_{2}$O 18 CF${}_{3}$(CF${}_{2}$)${}_{7}$(CH${}_{2}$)${}_{2}$OH	**4:1**	323	279.4
B16/10 1a	288 DPPC 8756 H${}_{2}$O 1 B16/10	**288:1**	330	50.0
B16/10 1b	288 DPPC 8756 H${}_{2}$O 1 B16/10	**288:1**	330	200.0
Pure 330	288 DPPC 8756 H${}_{2}$O	—	330	400.0
B16/10 6	288 DPPC 8756 H${}_{2}$O 6 B16/10	**48:1**	335	1000.0
Pure 335	288 DPPC 8756 H${}_{2}$O	—	335	400.0

**Table 2:** Self-diffusion coefficients of dipalmitoylphosphatidylcholine (DPPC) and additive molecules (assuming 2D diffusion; all simulations performed at 323 K and with 6:1 mixing ratio unless specified otherwise). Uncertainty given as 3σ (i.e., 99.7 % confidence interval) [31].

System	$D_{\mathrm{DPPC}}$ [pm${}^{2}$/ps]		$D_{\mathrm{Additive}}$ [pm${}^{2}$/ps]	
Pure	5.571	±0.219	—	
H10	11.983	±0.553	90.718	±6.702
H5F5	11.374	±0.267	48.369	±2.869
F10	1.116	±0.035	1.428	±0.136
FTOH	0.758	±0.032	0.739	±0.079
FTOH 333 K	7.091	±0.223	10.605	±0.757
FTOH 4:1	0.489	±0.022	0.480	±0.046
